# Endoscopic Gastroplasty in Obese Patients With a History of Cardiac Revascularization: A Retrospective Study

**DOI:** 10.7759/cureus.93300

**Published:** 2025-09-26

**Authors:** Dan Bandea, Thierry Manos, Marc Danan, Adrian-Valentin Cotirlet, Dan Dumitrescu, Bogdan Andrei Suciu, Anamaria Nedelcu, Ramon Vilallonga-Puy

**Affiliations:** 1 Doctoral School, George Emil Palade University of Medicine, Pharmacy, Science, and Technology of Târgu Mureș, Târgu Mureș, ROU; 2 Gastroenterology, Clinique Bouchard - ELSAN, Marseille, FRA; 3 Surgery, Clinique Saint-Michel - ELSAN, Toulon, FRA; 4 General Surgery, Moinesti Emergency Hospital, Moinești, ROU; 5 Surgery, University Emergency Hospital of Bucharest, Bucharest, ROU; 6 General Surgery, Carol Davila University of Medicine and Pharmacy, Bucharest, ROU; 7 General Surgery, Mures County Clinical Emergency Hospital, Târgu Mureș, ROU; 8 General Surgery, Clinique Saint-Michel - ELSAN, Toulon, FRA; 9 General Surgery, Autonomous University of Barcelona, Barcelona, ESP

**Keywords:** cardiac revascularization, class i, endoscopic gastroplasty, endoscopic plication, obesity

## Abstract

Background

Less invasive endoscopic bariatric procedures are being developed to manage Class I obesity. The most commonly used surgical techniques are laparoscopic sleeve gastrectomy (LSG) and Roux-en-Y gastric bypass (RYGB), with some cases utilizing adjustable gastric banding (AGB) or biliopancreatic diversion with duodenal switch (BPD-DS). This study aims to assess the efficacy of endoscopic gastroplasty (EG) in obese individuals with metabolic diseases, using an endoscopic suture, along with perioperative care and clinical outcomes over a 12-month follow-up period.

Methods

Eight patients were included in this retrospective study and underwent EG under general anesthesia with overnight hospitalization. We took into consideration elements such as changes in body weight and the occurrence of adverse events. Other studied parameters were demographic (age and sex), clinical (body mass index (BMI) and postoperative clinical manifestations), type of cardiac revascularization (CR), primary obesity or weight regain, and clinical follow-up. EG was recommended as an alternative to surgery due to personal choice or because of the risk of bleeding or cardiovascular events. Patients were divided into two groups to explore the correlation between BMI and potential factors contributing to enhanced weight loss after EG. Group A included four patients with a BMI < 35 and primary obesity or a history of gastric balloon placement, while Group B comprised four patients with a BMI > 35 or a history of bariatric surgery. All patients selected in the study underwent CR procedures prior to EG, such as percutaneous coronary intervention (PCI) or coronary artery bypass grafting (CABG).

Results

Endoscopic procedures were performed on eight patients with a mean age of 38.25 years for primary obesity or weight regain. The mean BMI was 34.5 kg/m², with a range between 30.2 and 42.4. No significant adverse events were observed during the endoscopic procedure or in the follow-up period. Seven patients (87.5%) experienced moderate post-procedural pain that lasted for an average of 6.5 days (range: 2-14 days), and five patients experienced nausea and vomiting, which improved with intravenous medications. Of the eight initial patients, seven were available for a three-month follow-up, six for a six-month follow-up, six for a nine-month follow-up, and four completed the 12-month assessment, with a mean excess weight loss (EWL) of 29%. We do not know the motivation for waiving postoperative follow-up except in one case - namely, a change of home address. In accordance with the American Society for Gastrointestinal Endoscopy (ASGE) criteria, two of the four 12-month follow-up patients achieved more than 25% EWL. One patient in Group A achieved successful weight loss with an EWL of 45%, whereas two-thirds of the patients in Group B failed to achieve an EWL greater than 25% at the 12-month follow-up. Additionally, all patients who had previously undergone bariatric surgery failed to achieve any significant weight loss after EG.

Conclusions

EG is a minimally invasive method with good tolerability. However, for patients who have had previous bariatric surgeries or those with severe obesity, the outcomes are suboptimal. Further studies involving larger cohorts and longer follow-up of obese individuals undergoing both CR and EG are needed to generate statistically significant data on post-procedural outcomes.

## Introduction

The National Institutes of Health (NIH) supports the use of weight reduction treatment for patients with a body mass index (BMI) of 35 or higher, as well as for individuals whose BMI ranges from 30 to 35, accompanied by a high-risk waist circumference and at least two additional comorbid risk factors, such as hypertension, type 2 diabetes, obstructive sleep apnea, or dyslipidemia [1].

Building on these guidelines, the 2022 recommendations from the American Society for Metabolic and Bariatric Surgery (ASMBS) and the International Federation for the Surgery of Obesity and Metabolic Disorders (IFSO) have further clarified the indications for metabolic and bariatric surgery. The new guidelines lower the threshold for surgery, recognizing it as a treatment option for patients with a BMI ≥ 35, regardless of the presence of comorbidities. Additionally, surgery is now recommended for patients with a BMI between 30 and 34.9 who have metabolic diseases, such as type 2 diabetes, even if their BMI is below the previous threshold [2]. These changes reflect a growing body of evidence demonstrating the metabolic benefits of bariatric surgery, especially in improving insulin sensitivity and reducing cardiovascular risk, even in patients with lower BMIs [3].

Individuals with Class III obesity (BMI ≥ 40) or those with Class II obesity (BMI ≥ 35) and major obesity-associated comorbidities, such as type 2 diabetes or obstructive sleep apnea, remain strong candidates for bariatric surgery [2].

The surgery has been shown to deliver substantial, long-term weight loss and marked improvement in obesity-related health conditions [2]. Importantly, the procedure not only aids in weight loss but also improves metabolic conditions by altering gut hormone levels, leading to better blood sugar control and reduced cardiovascular risks.

However, despite its substantial benefits, bariatric surgery is not risk-free. Although the perioperative mortality rate is low, patients must be aware of the potential long-term complications, such as nutritional deficiencies, dumping syndrome, and gastrointestinal issues. As emphasized in the updated guidelines, close postoperative follow-up is crucial to monitor for deficiencies in vitamins and minerals, and to ensure that patients adhere to necessary dietary and lifestyle changes [4,5]. In addition, the need for lifelong management, including regular follow-up visits with healthcare providers, is critical to maintain the positive outcomes of the surgery and prevent potential complications.

The updated 2022 ASMBS and IFSO guidelines reflect a more inclusive approach to bariatric surgery, broadening its indications to include patients with lower BMIs but other significant metabolic disorders [2]. This shift acknowledges the powerful metabolic effects of bariatric surgery, particularly in improving diabetes and reducing cardiovascular risks. Nevertheless, data on the risk of early complications (within 30 days) after bariatric surgery in patients with significant cardiovascular risk, particularly those who have undergone cardiac revascularization (CR), remain limited. These early complications generally fall into two primary categories: surgical complications, such as leaks and hemorrhages, and cardiovascular events, including myocardial infarction and arrhythmias. To address the heightened risks in this patient population, less invasive endoscopic procedures have emerged as viable alternatives. These options offer a safe approach to weight management for individuals considered too high-risk for traditional surgical interventions, expanding treatment possibilities for those with a complex medical background [2,5,6].

The endoscopic bariatric procedures are established as a valuable addition to the obesity treatment arsenal [6]. According to Kumar, endoscopic interventions surpass conservative measures in effectiveness and offer improved accessibility and reduced invasiveness relative to bariatric surgery [7].

The aim of this study was to evaluate our initial case series, focusing on the complications and short-term weight loss outcomes of endoscopic gastroplasty (EG) in a specific population of patients with a history of CR. This group had been previously assessed for bariatric surgery but was deemed high-risk due to concerns about bleeding and cardiovascular events. As a result, the less invasive endoscopic approach was prioritized for these patients.

EG has emerged as a minimally invasive option for the treatment of patients with failed or suboptimal bariatric procedures, such as gastric bypass or sleeve gastrectomy, by revising or tightening the gastric anatomy without requiring open surgery. In patients with previous CR, particularly those who have undergone procedures like coronary artery bypass grafting (CABG) or percutaneous coronary intervention (PCI), EG represents a unique solution [8-10].

This study evaluates the intersection of EG and patients with a history of CR, reviewing the safety and efficacy, perioperative management, postoperative complications, and follow-up for 12 months, with references to contemporary literature [11-14].

## Materials and methods

EG was performed on eight patients with prior CR at Bouchard Private Hospital - ELSAN, Marseille, France, between January 2021 and March 2025. Data on relevant parameters, such as clinical and demographic characteristics, type of CR, primary obesity or weight regain, and 12-month follow-up, were collected using the hospital’s electronic system, and key information was subsequently transferred to Microsoft Excel (Microsoft® Corp., Redmond, WA, USA). Eligibility for inclusion was determined according to the following criteria: individuals with previous CR, including different types of revascularizations (CABG, PCI, and CABG + PCI); obese patients (BMI = 30-40 kg/m²) who understood and committed to multidisciplinary follow-up for obesity for at least one year; individuals with a BMI exceeding 40 kg/m² who opted against bariatric surgery because of the perceived risk of complications; and individuals who had previously undergone bariatric surgery (laparoscopic sleeve gastrectomy (LSG) or Roux-en-Y gastric bypass (RYGB)) and opted not to pursue an additional surgical procedure.

EG contraindications are absolute and relative. Absolute contraindications include coagulation disorders (hemophilia and thrombocytopenia), active gastrointestinal inflammatory or bleeding lesions (Crohn's disease, ulcerative colitis, and gastric ulcer), gastrointestinal neoplasms, prior major gastric surgery that modifies the anatomy of the digestive tract, and anesthetic contraindications. Relative contraindications include esophagitis or gastroesophageal reflux disease, hiatal hernia greater than 3 cm, severe liver disease, untreated psychiatric disorders, addiction to prohibited substances, pregnancy, and the inability to follow the post-procedure program. The multidisciplinary bariatric team evaluates patients two months before the EG, and patients undergo upper endoscopy, abdominal ultrasound, nutritional and psychological counseling, anesthetic evaluation, and biological balance.

A 25% excess weight loss (EWL) at one year was recommended by the American Society for Gastrointestinal Endoscopy (ASGE) and the ASMBS task force as the benchmark for assessing the efficacy of endoscopic weight loss treatments [8]. After the endoscopic procedure, patients received intravenous analgesic and antiemetic medication, as well as proton pump inhibitors every 12 hours until symptoms resolved.

Technique description

The goal of EG is to reduce the gastric cavity to a tubular lumen by modifying the greater curvature with a row of tightened folds. This technique employs an endoscopic row of tightened gastric wall plications, resulting in a tubular gastric lumen approximating the shape of a sleeve gastrectomy without fully replicating its anatomical structure. The mechanism of action and gastric emptying time differ significantly from sleeve gastrectomy.

An endoscopic suturing device (OverStitch; Apollo Endosurgery Inc., Austin, TX, USA), attached to a dual-channel endoscope (GIF-2T160; Olympus Medical Systems Corp., Tokyo, Japan), is used to perform the procedure. The procedure is performed under general anesthesia with endotracheal intubation and with the patient in the left lateral position. A specially developed esophageal overtube (Apollo Endosurgery Inc.) is used to optimize procedural efficiency and enhance patient safety.

After the procedure is performed, a final gastroscopy ensures that the tubular configuration of the stomach is correct. It also examines whether there is a necessity to perform additional stitches and stops potential bleeding. The patient requires 24-hour surveillance in the immediate postoperative period. Eight hours after the intervention, an oral tolerance test is performed. Twenty-four-hour post-procedure blood tests are conducted to exclude the possibility of intraperitoneal hemorrhage. Post-procedural assessment of EG is performed at designated intervals using contrast-enhanced radiography.

For statistical analysis, continuous demographic variables are presented as mean ± standard deviation and range, while categorical variables and complications are reported as frequencies and percentages. Continuous outcome measures are also expressed as mean ± standard deviation and range. Descriptive statistics, including simple counts and mean values, are used to summarize complications and adverse events.

## Results

Endoscopic procedures were performed for primary obesity or weight regain. Most of the patients were female (n = 7), while one male patient (n = 1) was included in the study. The mean age was 38.25 years (range: 26-48), and the mean BMI was 34.5 (range: 30.2-42.4), as can be seen in Table 1.

**Table 1:** Age and BMI variation BMI, Body mass index

	Patient 1	Patient 2	Patient 3	Patient 4	Patient 5	Patient 6	Patient 7	Patient 8
Age (years)	33	26	37	34	41	48	45	42
BMI (kg/m^2^)	30.2	31.3	31	42.4	36	31.1	38	36

No significant adverse events were observed during the endoscopic procedure or in the follow-up period. Patients required hospitalization for a maximum of two days in the inpatient care unit. Two patients reported moderate post-procedural pain, and five patients needed adjuvant therapy for pain management for an average of 6.5 days (range: 2-14 days). Five patients experienced nausea and vomiting, which were alleviated by intravenous medications. Post-procedural complications were systematically evaluated daily by the attending physician and documented in the medical records using the numeric rating scale. The number of days during which complications such as pain, nausea, or vomiting occurred was quantified and summarized in Table 2.

**Table 2:** Numbers of days with post-procedural complications

	Patient 1	Patient 2	Patient 3	Patient 4	Patient 5	Patient 6	Patient 7	Patient 8
Pain	2	8	0	5	14	10	7	6
Nausea	0	0	0	2	3	3	2	3
Vomiting	0	0	0	1	1	1	1	2

Out of the eight initial patients, seven were available for a three-month follow-up, six for a six-month follow-up, six for a nine-month follow-up, and four completed the 12-month assessment. We obtained a mean EWL of 29% for individuals who completed the 12-month follow-up. Patients were divided into two groups to explore the correlation between BMI and potential factors contributing to enhanced weight loss after EG. Group A included four patients with a BMI < 35 and primary obesity or a history of gastric balloon placement, while Group B comprised four patients with a BMI > 35 or a history of bariatric surgery. In accordance with the ASGE criteria, two of the four 12-month follow-up patients achieved more than 25% EWL. Notably, the two patients who had previously undergone gastric bypass did not achieve any significant weight loss, with an EWL less than 25%, as shown in Table 3.

**Table 3:** EWL progression over the follow-up period EWL, Excess weight loss; RYGB, Roux-en-Y gastric bypass

Patient	Group	Prior Bariatric Surgery	3-Month EWL	6-Month EWL	9-Month EWL	12-Month EWL
1	A	Gastric Balloon	24%	36%	40%	45%
2	B	RYGB	11%	18%	20%	22%
3	B	None	6%	16%	20%	26%
4	B	RYGB	13%	19%	21%	23%

## Discussion

Nowadays, we are witnessing the emergence of a new direction in endoscopic intervention for patients with different types of bariatric surgery who have undergone CR. Less invasive endoscopic bariatric procedures are being developed to manage obese patients. This study aims to evaluate the effectiveness of EG in eight patients using a less invasive technique, as well as to assess perioperative management and outcomes throughout a 12-month follow-up period. Therefore, from the outset, we can conclude that more studies targeting EG in obese patients with previous CR need to be developed in the future.

Obesity is now recognized as a chronic disease with epidemic proportions and significant economic implications [15]. Many patients struggle for years with noninvasive measures like diet, exercise, and pharmacotherapy. Heart failure and prior CR can significantly impact the perioperative outcomes of bariatric surgery, raising concerns about the balance between potential benefits and risks in patients with multiple cardiac events [4]. For these high-risk individuals, the role of bariatric surgery needs to be reconsidered. EG offers a valuable alternative, filling the treatment gap by supporting patients in making the lifestyle changes necessary for long-term success [6,10]. A multidisciplinary team of bariatric specialists plays a crucial role in this process, providing continuous education, monitoring, and support [16]. In the present study, eight patients with a history of CR followed the same bariatric surgery protocol, both during the preoperative period and for one year of follow-up post-procedure.

CR procedures include CABG and PCI. CABG involves the creation of bypasses around occluded coronary arteries to improve blood flow to the heart muscle. PCI, often involving balloon angioplasty and stent placement, is used to open narrowed coronary arteries.

Both procedures improve myocardial perfusion but have long-term implications for patient management, particularly in relation to perioperative care and risk factors, such as antiplatelet therapy and anticoagulation.

In patients with CR, we must take into consideration the perioperative and postoperative risks. Patients with a history of CR are at higher perioperative risk for cardiovascular complications during any endoscopic procedure, including EG. The stress of any procedure can precipitate cardiac events, including myocardial infarction or arrhythmias. Therefore, close multidisciplinary collaboration between bariatric surgeons, gastroenterologists, and cardiologists is essential.

There are two aspects to consider: anesthesia and anticoagulant management. General anesthesia can raise significant risk, especially in patients with reduced left ventricular function, ongoing myocardial ischemia, or significant valvular disease. Non-invasive preoperative evaluation, such as stress testing or echocardiography, is often recommended. Patients with coronary stents, especially those on dual antiplatelet therapy (DAPT), present a challenge. Temporarily stopping treatment with antiplatelet agents increases the risk of stent thrombosis, while continuing treatment increases the risk of bleeding during EG. Guidelines often recommend bridging strategies with low molecular weight heparin (LMWH), or careful timing of the procedure [17]. In the first 30 postoperative days, our focus must be on cardiovascular, gastrointestinal, and nutritional monitoring. After EG, patients with a history of CR need continuous cardiovascular monitoring, as they remain at risk for complications such as myocardial ischemia or congestive heart failure exacerbation due to hemodynamic changes. Given that many patients with a history of CR may also have concurrent metabolic syndrome or diabetes, careful postoperative monitoring of blood glucose, lipid levels, and potential nutritional deficiencies is necessary to optimize outcomes.

EG has been criticized for not being sustainable. However, the first 12 to 18 months following bariatric procedures are marked by significant changes. EG is often preferred over a re-do metabolic surgery, particularly when the metabolic and cardiovascular burden is considered. Initial reports of EG demonstrated similar efficiency and durability [10-12]. Furthermore, EG has several important advantages: it does not cause irreversible anatomical changes in the gastric cavity, and the procedures are reproducible and repeatable, allowing future reinterventions to ensure sustainable results. While no major adverse events were observed in our study, the ability to reverse EG may offer a safety advantage if serious complications occur.

Evidence from our study suggests that EG is a safe and minimally invasive procedure with a low risk of complications. None of the eight patients in this group experienced bleeding or any other complications. However, in our previously published experience [14], we recorded two post-procedural complications out of 191 patients: one case involving a transparietal suturing of the falciform ligament, which required laparoscopic exploration due to severe abdominal pain, and another case of a perigastric collection, treated with antibiotics. Both cases had favorable outcomes.

A recent literature review [18] identified two cases of mortality due to hemorrhage following bariatric surgery [9,19]. While the data does not definitively indicate an increased risk of bleeding in this patient cohort, when hemorrhage occurs, the mortality risk is significantly higher within this group. This highlights the importance of considering alternative, less invasive approaches, such as EG - particularly for patients with increased cardiovascular risk - where the potential advantage of reducing surgical complications becomes even more critical.

While Espinet Coll et al. [20] described the potential for catastrophic hemorrhage and splenectomy when sutures are placed near the splenic hilum, the patients taken into consideration for this study did not face any significant complications. Lopez-Nava et al. [13] conducted a study in 2017 involving 248 patients treated with EG using the Apollo OverStitch system. Of the 248 patients, 2% (five individuals) experienced severe adverse events, yet none of these cases required surgical management.

Among the severe adverse events, two patients developed perigastric fluid collections, one experienced intra-abdominal hemorrhage successfully managed without surgery, another suffered a postoperative pulmonary embolism, and in one case both pneumothorax and pneumoperitoneum were involved, necessitating drainage. Based on the findings of that study, abdominal pain and nausea were the most prevalent minor complications, with reported incidence rates ranging from 27.5% to 80% and 38.5% to 80%, respectively. Patients received medication as needed.

A major limitation of our study is the relatively small sample size, which may affect our ability to detect statistically significant differences or rare complications that could emerge in a larger cohort. A further limitation of our study is the gender imbalance, as seven out of eight patients were female. The retrospective nature of the study and the short 12-month follow-up represent additional limitations. Despite this, the study still allowed us to thoroughly assess the safety profile of EG, particularly in relation to complications such as bleeding. Given that EG involves no resection of the stomach, the risk of bleeding is inherently lower compared to traditional bariatric surgery, which is a significant advantage for patients at higher cardiovascular risk [6]. One of the key strengths of this study is its novelty, to our knowledge, as it represents the first report on the use of EG in patients with a history of CR. The long-term significance of the procedure will be determined over time. This unique focus provides valuable insights into the feasibility and safety of EG as a less invasive alternative for a population that is often considered too high-risk for surgical weight-loss interventions.

## Conclusions

EG is a safe and minimally invasive method that proves its efficacy with good tolerability, particularly for primary obesity patients. However, for patients who have had previous bariatric surgeries or those with severe obesity, the outcomes are suboptimal. The multidisciplinary bariatric team plays a pivotal role in the selection and follow-up of obese patients. Further studies involving larger cohorts and longer follow-up of obese individuals undergoing both CR and EG are needed to generate statistically significant data on post-procedural outcomes, including both early and late postoperative complications. In patients who do not respond to treatment, additional investigations may be necessary to determine contributing factors outside the scope of this therapy.
